# Colour and pattern change against visually heterogeneous backgrounds in the tree frog *Hyla japonica*

**DOI:** 10.1038/srep22601

**Published:** 2016-03-02

**Authors:** Changku Kang, Ye Eun Kim, Yikweon Jang

**Affiliations:** 1 Department of Biology, Carleton University, Ottawa, ON, K1S 5B6, Canada; 2 Division of EcoScience, Ewha Womans University, Seoul, 120-570, Republic of Korea

## Abstract

Colour change in animals can be adaptive phenotypic plasticity in heterogeneous environments. Camouflage through background colour matching has been considered a primary force that drives the evolution of colour changing ability. However, the mechanism to which animals change their colour and patterns under visually heterogeneous backgrounds (i.e. consisting of more than one colour) has only been identified in limited taxa. Here, we investigated the colour change process of the Japanese tree frog (*Hyla japonica)* against patterned backgrounds and elucidated how the expression of dorsal patterns changes against various achromatic/chromatic backgrounds with/without patterns. Our main findings are i) frogs primarily responded to the achromatic differences in background, ii) their contrasting dorsal patterns were conditionally expressed dependent on the brightness of backgrounds, iii) against mixed coloured background, frogs adopted intermediate forms between two colours. Using predator (avian and snake) vision models, we determined that colour differences against different backgrounds yielded perceptible changes in dorsal colours. We also found substantial individual variation in colour changing ability and the levels of dorsal pattern expression between individuals. We discuss the possibility of correlational selection on colour changing ability and resting behaviour that maintains the high variation in colour changing ability within population.

In many animals, body colouration is primarily used for defence against predators^1^,^2^. Camouflage is the most commonly used and widespread defensive colouration. Predators will often fail to detect or recognize camouflaged prey^3^. There are different forms of camouflage that may reflect different protective mechanisms^2^,^4^, which include background matching^5^, disruptive colouration^6^, countershading^7^, or masquerade^8^. Among those, background colour matching (the minimization of colour difference between an animal’s body colouration and its background colours) is unarguably the most widespread in various taxa.

Camouflage through background matching can be achieved by possessing colours and patterns that resemble those of the animals’ background^3^,^9^. However, achieving background matching is complicated when the backgrounds are heterogeneous in colours and patterns. Natural backgrounds often exhibit substantial spatial and temporal variation in both colour and pattern^10^,^11^,^12^. In turn, some organisms have evolved body colouration plasticity, so called colour change, in order to cope with heterogeneous backgrounds^11^,^13^.

Colour change is found in a variety of taxa including fish^14^,^15^, amphibians^16^, crustaceans^17^, and insects^13^. Anurans are one of the most intensively studied organisms for colour change, and the proximate mechanisms that mediate the colour change in anurans are a century old question that is still under investigation^16^,^18^,^19^. Physiologically, anuran colour change occurs due to the synchronous movements of pigment organelles within chromatophores, which are largely regulated by hormones (for the detailed mechanisms, see Nielsen^20^). External environmental factors for colour change in anurans include light intensity, background colour, and temperature^21^. The evidence of frogs’ response to light intensity and temperatures align well across many studies and concludes that frogs become brighter under stronger illumination and higher temperature^22^. Temperature-dependent colour changes have also been considered an adaptation for thermoregulation since darker colours are more likely to absorb solar energy^21^,^23^.

Colour changing animals are able to change their colours or patterns to enhance the degree of camouflage (e.g. background colour matching or disruptive pattern expression) against their background^24^,^25^. In anurans, the evidence of camouflage in terms of both achromatic and chromatic colour matching are present^21^,^26^,^27^. However, it has rarely been tested how animals change their colours under the conditions of mixed-colour backgrounds which can be also found in many natural substrates (but see Kats and van Dragt^23^). Colour changing animals provide good opportunities to explore how animals adopt/optimize their colours and patterns for camouflage when a background consists of more than one colour. We predict that colour changing animals would exhibit either of the two strategies against mixed-colour backgrounds: ‘intermediate’ colour between two background colours or a specialized colour that specifically matches either background colours^28^,^29^.

Although most of the experiments with frogs tested the change in colours, colours are not the only properties that can change in anurans^30^. Frogs can change the expression of dorsal patterns and body colouration, often simultaneously^31^. This simultaneous change is pronounced in tree frogs since the dorsal pattern expression varies from complete disappearance to highly contrasting patterns within individuals. These patterns may improve camouflage by either the disruption of edges^6^,^32^, and/or background pattern/complexity matching^10^,^33^.

Physiological colour changes in anurans are predicted to occur rapidly^20^,^34^. However, there still exists evidence for both short-term (from minutes to hours^19^,^23^) and long-term colour change (several days^26^,^27^,^35^). The evidence of long-term colour change has been reported under a constant environmental condition throughout the experimental days^26^,^27^. However, because the external conditions (e.g. temperature, light intensity, background colour) of a frog change within/between days in natural circumstances, whether the long-term colour change occurs in natural conditions is not understood well. The most abrupt change in external conditions that might interfere the long-term colour change process would be the change in light intensity between day and night. The exposure to darker and duller conditions during a night could mean that long-term colour changing processes are less likely to occur, if in fact the period of darkness “resets” the colours of the frogs to a dark and dull colour every night^22^. Therefore, it is crucial to examine how frog colour changes at night when the light intensity and exposure decreases dramatically.

Some animals, such as cuttlefish, can actively modulate their body colours through visual perception of the background^24^, but it has rarely been demonstrated in other taxa (including anurans as far as we know) whether the animal’s visual perception of environments plays role in colour change processes. However, if colour change evolved to increase the efficacy of the visual signal, the more important factor that may drive the colour change ability would be the visual systems of the receivers. In terms of camouflage, the receivers are the animals’ natural predators. Although the evidence of camouflage function of colour change (through background colour matching) in frogs are plentiful, no studies have considered the vision of their natural predators^9^,^27^. To completely understand the adaptive significance of colour change, it is crucial to understand whether this change is being perceived by natural predators^36^.

In this study, we investigated the colour change of the Japanese tree frog, *Hyla japonica* (Hylidae; Günther, 1859), against heterogeneously coloured backgrounds. We mainly examined how the frogs’ colour and dorsal patterning changed against differently coloured backgrounds. We estimated whether the differences in dorsal colour against different backgrounds were large enough to improve camouflage from natural predators’ point of view. Furthermore, we tested whether colour change on mixed coloured backgrounds was an intermediate body colour or matched with one of the background colours.

## Methods

### General experimental design

We collected male *H. japonica* from agricultural fields and rice paddies near Mt. Bukhan (37.6 °N, 126.9 °E). Catching/housing of frogs, and the experimental procedures were approved by Ewha University Institutional Animal Care and Use Committees (IACUC 2013-01-084) in accordance with the approved guidelines. Due to the low abundance of females, males were chosen for the experiment. The collected frogs were kept individually in small containers and transported to a temperature and humidity controlled room (25 ± 2 °C, 75 ± 5%) at Ewha University. Each frog was provided with two juvenile crickets as food source per day and a small petri dish with a thin layer of water. Frogs were housed in small cube-shaped cells (0.5 × 0.5 × 0.5 m) that were independently illuminated by a daylight LED bulb (POSCO 9W 5000°K, Yong-in, South Korea; see Supplementary Fig. S1 for spectral properties) from the ceiling. Photoperiod was 12:12 L:D.

During the colour changing trial, we kept each frog in a cylindrical container, which was located in the cube-shaped cell, with the top covered by a transparent glass (which were removed when taking photos) through which light can reach inside the container. The inside of the container was covered by printed papers that provided various background colours. To ensure equal illumination, we adjusted the height of bulb such that illuminance was 100 lux at the centre of each container.

Because frogs can rapidly change dorsal colours under stress or during handling, non-invasive methods are preferred for quantification of frog colouration^20^. Photography of frogs is a method of colour quantification that limits direct contact with frogs and allows post-processing^37^. The issue with digital photography is that most commercial cameras do not capture light within the UV range (300–400 nm) that can be perceived by natural predators of frogs^36^. However, this problem is of less concern if an animal and its natural backgrounds reflect low levels of light in the UV region^37^,^38^. We checked for the UV reflection in frogs (N = 14) using a spectrometer (JAZ, Ocean Optics, Dunedin, Florida, USA) and found negligible UV reflectance (Supplementary Fig. S2). Because most of their natural backgrounds such as leaves, soil, or tree barks also reflect low levels of UV, we considered that photographic method can capture full spectrum range of colour change in *H. japonica*.

The colour-change trial began at 8:00AM (UTC+09:00) every day in June and July of 2014. Because the photophase always started at 8:00AM, the potential effect of circadian rhythms on the expression of colours between treatments would be minimized^39^. A frog was tested only in one type of background each day. Prior to each trial, each frog was transported to a pre-assigned coloured container (see below for the colour assignment) and waited under darkness. Subsequently, we photographed each frog at 0, 1, 2, 4, 8, 12, and 23 h after the light was turned on (turned off at 12 h). Hereafter, duration referred to the amount of time that a frog was exposed under a background type. For each duration, photography was done in the photographic arena. The photographic arena had the same setup as the cell where colour change trials were conducted, but a camera was set next to the bulb side by side to photograph the frogs’ dorsal colour from above. The photography process usually took less than a minute for each frog. After 12 h, they remained in the same container, spent the night under darkness, and were photographed at 23 h (7 am next day) to document the degree of colour change under night-time. Then, between 7 am and 8 am, they were transported to the next container with a different background and waited for the initiation of the next trial. We used a Sony α65 camera equipped with SAL1855 lens (Sony, Japan) for photography. We used a constant exposure setting throughout the experiments. Every morning, just before the initiation of each trial, we took photos of X-rite colour checker (Grand Rapids, Michigan, U.S.) inside of each coloured container and used the six grey colours of the colour checker for image calibration (see below). After the experiments, frogs were released at their captured sites.

### BW experiment

**Figure 1:**
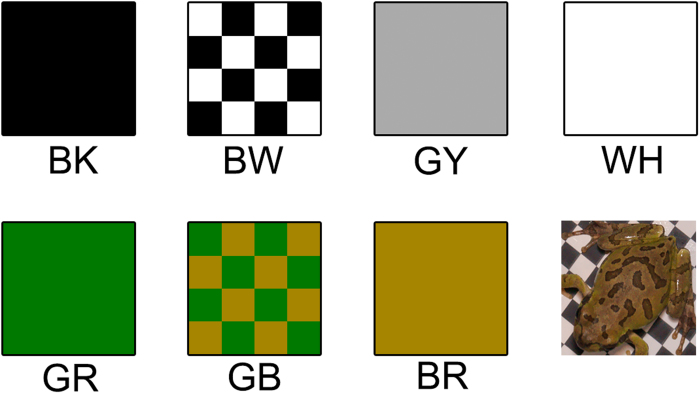
Background colours that were used for BW (upper panels) and GB (lower left three panels) experiments. Black (BK), black/white check-patterned (BW), grey (GY), white (WH), green (GR), green/brown check-patterned (GB), and brown (BR) background. The photo in the lower right side is an example of *H. japonica* with contrasting dorsal patterns against BW background.

In this experiment, we tested how the colours and patterns of frogs change against four achromatic background colours (Fig. 1 upper panel, see Supplementary Fig. S3 for reflectance data): black (BK), white (WH), grey (GY), and black-white check pattern (BW). The background colours were printed on waterproof papers (HP Laserjet Tough Paper) with a printer (Colour LaserJet 5550; Hewlett-Packard; Palo Alto, CA, USA). For BK and WH backgrounds, we printed black and white colour (R = G = B = 0 for BK colour, R = G = B = 255 for WH colour) using Photoshop (Adobe Systems Inc.; San Jose, CA, USA). For GY background, we were unable to find a colour that showed flat reflectance across whole wavelength range (400–700 nm). Instead we printed out one of the grey colours (i.e. the colour with R = G = B) that showed an intermediate reflectance between WH and BK colour within red-green colour region (550–700 nm) because frog colour change were expected to be most prominent within this wavelength region. For BW background, we generated black/white check pattern with 5 mm interval. This pattern size was chosen because 1) the square area of each pattern was considerably smaller than the size of frogs so that frogs were always on a mixture of white and black patterns, and 2) the area of square pattern is comparable to the patterns on frogs’ dorsa (see Fig. 1). We note here that the reflectance of BK colour was near 0 across all wavelengths, but WH/GY colour showed higher reflectance near blue colour region (400–550 nm) which is the properties of most commercial white papers. Therefore, although we consider that most of the colour difference between each background in BW experiment accounted for the differences in achromatic properties, there were small levels of uncontrolled chromaticity in GY and WH colours (BK: brightness = 6.67, chroma = 0.45, hue = −1.23; GY: brightness = 73.31, chroma = 4.42, hue = −1.36; WH: brightness = 100, chroma = 6.2, hue = −1.32 after printing; the brightness of GY colour was brighter than 50% of WH colour because of the higher reflection in 400–550 nm region.).

Each frog (N = 48) was tested on all four backgrounds and the order of four-colour trials followed 4 × 4 Latin Square design for every four frogs.

### GB experiment

In this experiment, we used additional 24 frogs (N = 24; distinct from BW experiment) and examined the colour and pattern change in frogs against three chromatic backgrounds with similar brightness (Fig. 1 bottom panel): green (GR), brown (BR), and green-brown check pattern (GB). Green and brown colours are more representative of natural backgrounds such as leaves or leaf litter . Males of *H. japonica* are known to rest on leaves or within leaf litter, whose primary colours were dominated by green and brown during daytime (A. Borzée, unpublished). The properties of printed colours are dependent on the RGB values of pixels and printer properties. To print the colours that imitate the chromaticity of natural objects, colours required calibration. To do so, a colour palette (comprising of 2040 different colours from the web) was printed and photographed with five fresh and five dead leaves in a single shot. We then searched for the colour with the most similar RGB values to the averaged RGB values of both leaf types. The chosen green and brown colours were clearly different chromatically, but were similar to each other in terms of brightness (GR: brightness = 24.31, hue = −0.37, chroma = 20.58; BR: brightness = 27.02, hue = 0.74, chroma = 31.68 after printing). The visual system of *H. japonica* is unknown. Based on the known spectral sensitivities of *H. cinerea*^40^ and animal vision modelling^41^ (described in Supplementary methods in detail), however, GR and BR colours may be discriminable from the vision of *H. japonica* (just noticeable difference >5; just noticeable difference is a contrast estimate between two colours from an animal’s point of view, see below).

We did not use the background with intermediate hue value that was equivalent to grey background in BW experiment because, unlike achromatic backgrounds that have almost flat reflectance values across visible wavelengths which makes it possible to have average brightness, hue is a qualitative trait by which averaging results in different colours dependent on the types of colour space^42^. Each frog was tested on all background types and the order of backgrounds followed a 3 × 3 Latin Square design for every three frogs. At completion of the GB experiments, frogs were additionally tested on BK and WH backgrounds to estimate colour changing capacity and pattern expression (see below).

### Image analysis

Images were taken as a RAW format and converted to the tiff format using Image Data Converter (Sony, Japan). Then, we cropped each image to only have frog body and reduced to 600 × 600 pixels using bicubic interpolation. To remove non-linearity of camera responses, we performed linearization processes using grey colours in the colour checker that were photographed each morning^37^. Then each colour channel was equalized by scaling to the RGB value of grey^37^. After this processing, the RGB values of the processed images represented physical properties of the colours, independent of device and light conditions, and represented the reflectance of each pixel ranges from 0–255, corresponding to the reflectance of 0–100%.

For each image, we categorized whether the frog’s dorsal pattern was visible or not as presence or absence. Then, we randomly selected three regions of interest (ROIs) from the dorsum of frogs and measured median RGB values of the ROIs. The presence of dorsal patterns was often dependent on the background type even within individuals. Therefore, to properly compare how frog colours changed independent of the pattern appearance, we only selected basal region (outside of the pattern) for colour comparison. When frogs were patterned, the inside of a pattern was always darker than the outside basal regions (Fig. 1 bottom right panel).

The modelling of predator vision requires assuming specific predator species since the visual systems of predators are distinct to each other^41^,^43^. However, because tree frogs are preyed upon by a variety of predators including birds, snakes, or mammals, it is difficult to assume one predator type^44^. Here, we primarily used Lab colour space to analyse colours of each frogs^42^. Lab colour space is designed to approximate human vision, and one of the advantages of using this colour space is that it has perceptually uniform space: the Euclidean distance (∆E) between two colours reflects the perceived difference in human vision^42^. We converted measured median RGB values to Lab values and derived brightness, hue, chroma of each frog’s dorsal colour based on following equations^42^:


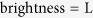



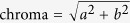



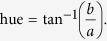


Once we detected changes in either of these colour properties against different backgrounds, we transformed the image’s red, green, and blue channel pixel values to a bird-specific colour space (predicted photon catches for each cone cell type) using a polynomial mapping method^37^,^45^ by a custom-built MATLAB program. We derived the contrasts between i) background colour and frog dorsal colour and ii) between individual frog’s dorsal colour against different backgrounds using receptor-noise-limited visual discrimination model^41^. This model produces both chromatic and achromatic contrasts as a unit of just noticeable difference (JND) between two colours in the receptor space. When JND ≥1, the difference between two colours is considered to be noticeable to tree frogs’ natural predators. The references for avian vision were the blue tit (*Cyanistes caeruleus*) single (chromatic) and double (achromatic) cones, and the references for snake vision were the garter snake (*Thamnophis sirtalis*) single cones (chromatic) and long wavelength sensitive single cones (achromatic)^46^,^47^. Because both background and frog body did not reflect significant UV lights (Supplementary Figs S2, S3), we assumed that photon catches from UV-sensitive cone cells were 0. The detailed explanation of predator vision modelling can be found in the Supplementary Methods and elsewhere^41^. ImageJ (NIH, Bethesda, MD, USA) was used for the image analysis.

### Data analysis

First, we examined the speed of colour change. We calculated the ∆E of the colour of frogs at each duration from the initiation of each trial. Then we compared the differences in ∆E between each duration using generalized linear mixed models (GLMMs) using the colour of current background and previously presented background as covariates, frog id as a random factor. Based on the result of this analysis, we used the frog colour that had completed colour changing process (two hours after being against one background; see results) as a representative colour against each background for further analysis. We further evaluated whether the frog colour after two hours against current background was independent of the colour of the previously tested background using MANOVA by putting brightness, chroma, hue at two hours as dependent variables, the colour of current background and previous background as independent variables.

Within individual frogs, colour difference was most prominent between BK and WH backgrounds (see results). To estimate individual frog’s capacity to change colour, we measured the ∆E between the frog’s colour against BK and WH backgrounds and used this distance as an index of individual colour change capacity.

To compare frogs’ dorsal colours between different background types, we examined within-subject effects of background type on brightness, chroma, and hue, using repeated measures multivariate analysis of variance. We then performed post-hoc multiple comparisons for the colour components that had significant effects. P values were adjusted to control for the false discovery rates^48^. To elucidate background colour matching function from the viewpoint of predators, we compared whether JNDs were lowest when the background-frog colours were matched (e.g. a frog’s colour against BK background yielded lowest JNDs with BK colour rather than GY and WH colours) using GLMMs.

In addition, we analysed the effects of background type on the presence of dorsal pattern using GLMMs to examine whether dorsal pattern appearance was affected by background colour. To identify the magnitude of colour change under darkness during night, we compared the colours of frogs just before becoming dark (at 12 h) with the colours at 7 am next day using repeated measures analysis of variance.

For all individuals tested (N = 72), we characterized the individual capacity to change colour and the exhibition of dorsal patterns. Since these two characteristics were assumed to be physiologically connected between each other, we examined the relationship between colour changing capacity and the ability to exhibit dorsal patterns. First, we categorized each frog into a ‘patterned’ or ‘non-patterned’ group based on whether they exhibited dorsal patterns against BK background. We then employed a logistic regression to analyse the relationship between colour changing ability and the ability to exhibit dorsal patterns. All the statistical analysis were two-tailed and conducted in R.

## Results

### Colour change speed and capacity

**Figure 2:**
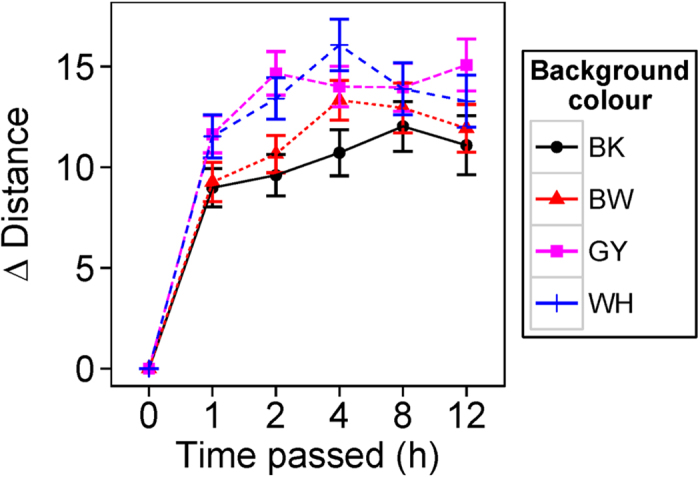
The relationship between time passed since the initiation of each colour changing trial and the Euclidean colour distance (∆E) from the initial status in BW experiment (N = 48). In all four types of backgrounds, frog colours changed rapidly within one hour and maintained similar levels throughout the remaining time. Symbols represent mean values and bars denote standard error of the mean.

In the BW experiment, the dorsal colours of frogs changed with time and duration was a significant factor for colour change (Fig. 2; χ^25^ = 595.56, P < 0.001). We found that the dorsal colours of frogs changed rapidly within an hour of trial initiation and maintained similar levels throughout the daytime (Fig. 2). Post-hoc comparisons suggest that there were no significant changes in ∆E after two hours, which was also the trend for the GB experiment (Supplementary Fig. S4). We also found no effect of the colour of previously tested background on the frog colour at two hours (Wilk’s λ = 0.89, approximate F_9,338_ = 1.29, P = 0.22). This suggests that frog colours at two hours were independent of the colour of previously tested background. Therefore, we used the colour of frogs at two hours as a representative colour on each background for further analysis. By using the colour of frogs that completely had undergone colour change process against their current background, we consider that further results are independent of the colour of the previously tested background.

We found substantial differences in colour changing ability between the frogs. In a pooled group of frogs from both BW and GB experiments, the distribution of colour changing capacity followed unimodal distribution (Supplementary Fig. S5; D = 0.03, N = 72, P = 0.88), which indicates that colour changing ability is a continuously varying trait in *H. japonica*, unlike *H. regilla* that shows discrete variation in colour changing ability^30^.

### BW experiment: brightness, chroma, and hue

**Figure 3:**
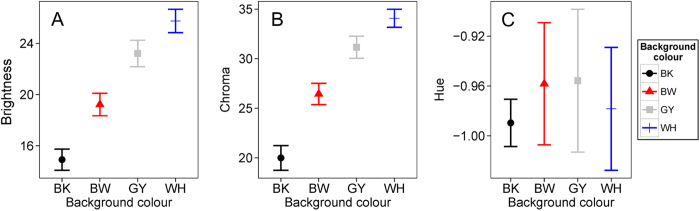
(**A**) Brightness, (**B**) chroma, and (**C**) hue of dorsal colour of frogs against each achromatic backgrounds (N = 48). Symbols represent mean values and bars denote standard error of the mean.

We found that the colour components were significantly different between background types (Fig. 3; Wilk’s λ = 0.51, approximate F_9,338_ = 12.00, P < 0.001). Univariate analysis revealed that frogs’ dorsal colour changed in both brightness (Fig. 3A; F_3,141_ = 31.58, P < 0.001) and chroma (Fig. 3B; F_3,141_ = 37.98, P < 0.001), but not in hue (Fig. 3C; F_3,141_ = 0.14, P = 0.93). Post-hoc tests revealed that both frogs’ dorsal brightness and chroma were different between all four background types (Supplementary Table S1). Brightness and chroma varied between WH, GY, BW, and BK background (highest to lowest; Fig. 3). Brightness and chroma against BW background showed intermediate values between WH and BK background, which indicates that frogs adopted intermediate forms when their backgrounds consisted of two colours.

**Figure 4:**
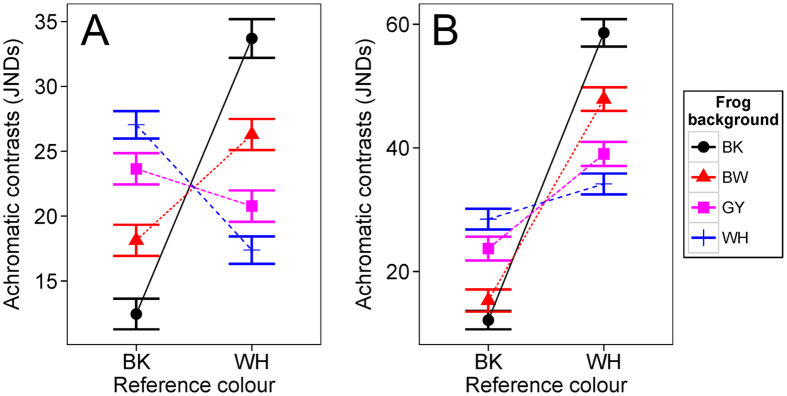
Achromatic contrasts between reference colours and frogs’ dorsal colours against each background type. Reference colours were black (BK) and white (WH). We derived just noticeable differences (JNDs; see methods) from (**A**) avian and (**B**) snake vision model. A frog may be detected by the predator when JNDs >1. Symbols represent mean values and bars denote standard error of the mean.

Estimated JNDs suggest that achromatic differences between frogs against different backgrounds were all discriminable (all JNDs >4) to both avian and snake predators (Table 1). Frogs tested on BK backgrounds had significantly lower JNDs from BK colour than any other backgrounds while frogs tested on WH backgrounds had significantly lower JNDs from WH colour (Fig. 4; avian model: χ^2^_6_ = 245.02, P < 0.001; snake model: χ^2^_6_ = 186.15, P < 0.001; see Supplementary results for full comparisons). These support the camouflage function of colour change in *H. japonica*. We presented here only achromatic JNDs from BK and WH colour, but not GY colour because brightness of GY and WH were not significantly different for both avian and snake models presumably because the GY colour that we chose for the experiment was already highly bright (because of the higher reflection in 400–550 region, see Supplementary Fig. S3) from the perspective of predators. Although we used GY colour that showed an intermediate reflectance between WH and BK within red-green colour regions, frogs’ responses to background seemed to be affected by the overall brightness of colour across whole wavelength range. This might be one potential reason why we find higher brightness in frog colours against GY than BW background.

In terms of chromaticity, the colours of frogs in different backgrounds were all discriminable to predators between each other (Table 1). However, when we estimated the contrasts between current background and frog dorsal colour and compared them between backgrounds, we found no evidence that frogs’ chromaticity matched better against the tested background than other backgrounds from the viewpoint of avian (χ^2^_6_ = 0.01, P = 1) and snake predators (χ^2^_6_ = 0.00, P = 1). This indicates that, although the chromaticity of frog dorsal colour changed dependent on the background type, it did not achieve any better colour matching in terms of chromaticity.

### BW experiment: dorsal pattern visibility

**Figure 5:**
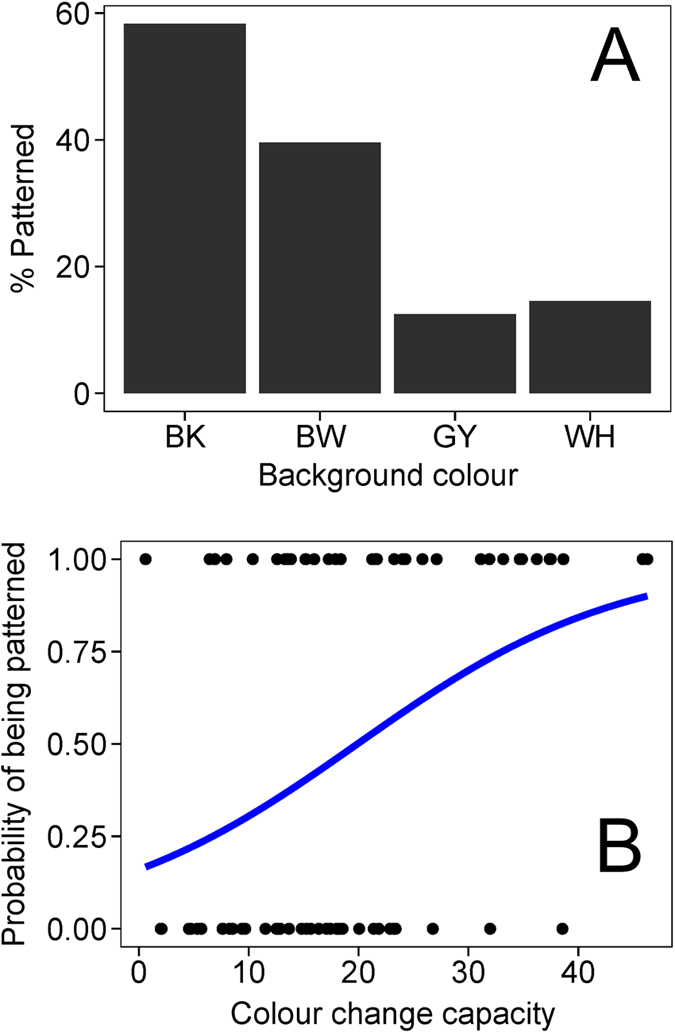
Proportion of patterned individuals against each achromatic background (A; N = 48) and the relationship between individual colour changing capacity and the presence of dorsal patterns against BK background (B; N = 72). (**A**) Frogs exhibited dorsal patterns more often when they were against darker backgrounds rather than brighter backgrounds. (**B**) Frogs with higher colour changing capacity were more likely to exhibit dorsal patterns.

We found substantial within-individual variation in the expression of dorsal patterns against differently coloured background (Fig. 5A; χ^2^_3_ = 21.08, P < 0.001). Dorsal pattern expression under BK background was significantly higher than any other background types (BK vs. BW: Z = −2.49, P_adj_ = 0.02, BK vs. GY: Z = −4.26, P_adj_ < 0.001, BK vs. WH: Z = −4.208, P_adj_ < 0.001). They also exhibited dorsal patterns more often under BW background than both GY (Z = −3.32, P_adj_ = 0.002) and WH (Z = −3.16, P_adj_ = 0.002) backgrounds. There was no difference in dorsal pattern expression between GY and WH backgrounds (Z = 0.43, P_adj_ = 0.67).

### BW experiment: colour change under darkness

**Figure 6:**
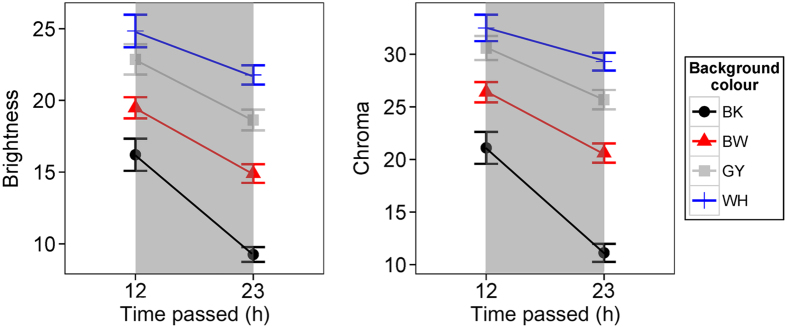
Colour change of frogs during night-time under darkness in BW experiments (N = 48). Frogs retained substantial colour properties of the previous day in terms of both (**A**) brightness and (**B**) chroma. Symbols represent mean values and bars denote standard error of the mean.

During night-time under darkness, colour properties changed substantially (Wilks λ = 0.51, F_9,796_ = 28.52, P < 0.001) depending on the type of backgrounds (Wilks λ = 0.80, F_3,327_ = 27.98, P < 0.001). We also found significant interactions between colour component and background type (Wilks λ = 0.93, F_9,796_ = 2.76, P = 0.003). Univariate analysis of each response variable showed that the frogs became darker and less colourful in all colour treatments (Fig. 6; brightness: F_1,329_ = 70.57, P < 0.001; chroma: F_1,329_ = 69.71, P < 0.001), but both brightness and chroma after the night were significantly affected by the background colour that they were being tested the previous day (Fig. 6; brightness: F_3,329_ = 67.09, P < 0.001; chroma: F_1,329_ = 81.88, P < 0.001; brighter and more colourful on WH, GY, BW, and BK background (highest to lowest)). We found no significant interaction between duration and background colour in brightness comparisons (F_3,329_ = 2.13, P = 0.10), but a single significant interaction was detected in chroma comparisons (F_3,329_ = 4.06, P = 0.007). We found no differences in hue analysis (all P>0.05). These collectively imply that, after exposed under darkness during a night, frogs became darker and less colourful, but largely retained brightness and chroma from the previous day especially when they were against brighter backgrounds (Fig. 6).

### GB experiment: overall results

We found no differences in brightness, chroma, and hue of frogs’ dorsal colours between all backgrounds (all P>0.65). However, we note here that, although obscured by group effect, two out of 24 frogs showed clear hue change from green to brown (Supplementary Fig. S6; green on GR background and brown on BR background). The effect of background colour was not significant for the expression of dorsal pattern (χ^2^_2_ = 0.64, P = 0.73; 29%, 25%, 25% for GR, GB, BR background respectively).

### Relationship between dorsal pattern expression and colour changing capacity

The above results suggest that individual frogs have different levels of colour changing ability as well as dorsal pattern expression. Against BK background, the dorsal patterns appeared in 34 out of 72 frogs. Logistic regression analysis showed that frogs with higher colour changing ability were more likely to express dorsal patterns (Fig. 5B; *Z* = 3.02, *P* = 0.003).

## Discussion

### Colour change against uniform vs. heterogeneous backgrounds

Our results indicate that, on average, frogs’ response to mixed colours followed an intermediate form rather than specifically matching one of the colours when they were against patterned background^49^. In addition, we found that there exists within population variation in colour changing ability: the degree of colour change was almost negligible (∆E ≈ 0) for the individuals with low colour changing ability (hereafter, non-changers), but colour change was considerably higher for the individuals with high colour changing ability (colour changers; Supplementary Fig. S6 for the distribution). Although the distribution of colour changing abilities has continuous distribution so that it is difficult to categorize them as two distinct groups, we contend that non-changers adopted the specialized camouflage strategy that only matched a background type while colour-changers adaptively changed their dorsal colours based on background brightness, and adopted the intermediate forms when they were against mixed coloured background^26^,^49^. We note here that the speculation that non-changers adopt specialized camouflage strategy only makes sense when non-changers indeed rest on one type of background that has the similar colour to their dorsal colour. Therefore, we encourage future experiments to test the resting preference of non-changers using natural substrates to evaluate this speculation.

### Individual variation in colour change capacity

What maintains this intra-population variation in colour changing capacity? Obviously, colour changing ability would be advantageous in terms of camouflage, since they can achieve better background matching against various substrates. However, this plasticity in dorsal colour expression can have costs, such as physiological costs to rearrange numerous pigments in chromatophores^50^,^51^. Therefore, there may be opposing selection pressures for higher phenotypic plasticity and lower energetic costs that would affect the phenotypes and behaviours of frogs.

It has been hypothesized that the variation in colour changing ability in frogs can be maintained by the correlational selection between microhabitat (background colour) preference and colour changing ability. Non-changers would prefer to stay on a uniformly coloured substrate that have similar colours of their dorsal colours while colour-changers would not have such preferences^52^. Background selection experiments revealed that *H. regilla*, especially non-changers, prefer to stay against the background that exhibits similar colours of their dorsal colour^52^. In natural circumstances, behavioural preference for colour matching background does not assume visual recognition of colours, but colour matching can also be achieved by using other correlated visual/non-visual cues^53^. For example, preferences for resting on the ground or leaves may not necessarily mean frogs rely on the colour cues but rather other sensory cues, such as shape or location of the substrates, which correlate with their colours in nature. These hypotheses are based on the assumption that colour change ability has genetic basis. However, our current understanding on how colour change ability of an individual is determined is limited, and we have no evidence that whether this plasticity is induced by genetic or environmental factors. Therefore, we encourage prospective experiments to explore genetic basis of colour changing ability through selection or cross fostering experiments. Then, the examination of substrate-frog colour matching under natural environments and identifying the sensory basis of it would also give us a new insight on how this plasticity can be maintained in a population.

### Dorsal patterns

Patterns are important elements for camouflage because they can reinforce the camouflage of animals by providing either background resemblance, disruption effects that hinder the detection of true outlines of frogs^5^,^6^, or increasing the complexity of animals which makes predators difficult to detect camouflaged targets^10^. In our study, *H. japonica* exhibited dorsal patterns considerably more often against darker background while only about 15% of the frogs exhibited dorsal patterns against brighter backgrounds (white and grey). Although the frogs exhibited dorsal patterns more often against check patterned background than brighter backgrounds, it seems to be driven by the overall brightness of the background, not by the existence of background pattern: the proportion of patterned individuals against pattered background were about the half between those against black and white backgrounds. Therefore, we suggest that dorsal pattern expression in *H. japonica* was affected by overall background brightness, not by the presence of background pattern. Here, we propose an adaptive hypothesis for the observed conditional expression of dorsal patterns.

If we apply our results to natural substrates, substrates that exhibit brighter background colours would be leaves while darker substrates would be tree bark or leaf litter. In general, leaves usually are homogenous in colour and are rarely patterned, whereas tree bark and/or leaf litter are often found with complex patterns. Therefore, considering the presence of patterns are likely to be advantageous against patterned background, having patterns against tree bark or leaf litters may be advantageous for frogs, while having patterns against leaves would be detrimental for survival. However, we cannot exclude the possibility of alternative non-adaptive explanations such as dorsal patterns as a by-product of melanophore re-arrangement during colour change process.

Our results also suggest that this conditional expression of dorsal patterns is more pronounced for colour-changers. Therefore, colour-changers would be benefited not only by their high colour changing ability, but also by their ability to express dorsal patterns against dark heterogeneous substrates. This further highlights the pressure for the correlational selection between microhabitat preference and colour changing ability. It would be advantageous for the non-changers to preferentially rest on one substrate (lighter substrate such as leaves) because they are less likely to exhibit dorsal patterns against darker substrates. The mean brightness of non-colour changers in our experiments (individuals with ∆E < 8, N = 13, mean brightness = 21.20) was comparable to the brightness of naturalistic green colour that we used (mean brightness = 24.31) which supports this prediction.

### Brightness, chroma, and hue changes

Our results corroborate the prediction of the physiological models of colour change in that two colour properties, brightness and chroma, were responsible for dorsal colour change in tree frogs^54^. These changes are readily perceivable to avian and snake predators in terms of both colour and brightness, which strongly supports that colour change evolved and was maintained because of its survival advantage through better background matching. Ultrastructural changes in pigments during colour change process are well documented in *H. arborea*. We will not discuss the details of the complex pigment rearrangement here (see^54^), but one of the main changes during a colour change from a light to a dark colour is that the melanosomes of melanophores disperse and surround chromatophores such as iridophores and xanthophores. Therefore, it is predicted that the change in brightness would also be accompanied by the change in chroma. In our experiment, brightness and chroma of the frog dorsal colour changed correlatively (the increase in brightness resulted in the increase in chroma; Supplementary Fig. S7) which supports this prediction. Although green and brown colours chromatically mimic naturalistic backgrounds in the wild, frogs exhibit similar colours against the two backgrounds. This indicates that the dorsal colours of frogs mainly responded to the achromatic properties of background rather than chromatic properties. It has been largely assumed that avian predators primarily use achromatic contrast (i.e. contrast in brightness) for the discrimination of small objects^55^,^56^. Therefore, to make predators fail to discriminate frogs against background, background matching in terms of brightness would be more important than chromaticity matching.

In BW experiment, GY colour represented the colour that showed intermediate reflectance between WH and BK colour within red-green colour region, but had higher brightness within the whole visible wavelength range. The fact that frogs adopted brighter colour against GY background than BW background implies that their responses to background colour were affected by the overall brightness of visible light rather than the brightness of red-green wavelength regions. We note here that our choice of bright GY background only affected frogs’ colour expression against GY background, but does not qualitatively change any other main findings of this study.

Although our sample sizes are not sufficient enough for the firm conclusion, about 10% of the frogs that were tested in GB experiments were able to change hue in response to background hues (Supplementary Fig. 6). This suggests the existence of polymorphic forms of *H. japonica* in terms of hue changing ability similar to the polymorphic forms of *H. regilla*^26^. It would be important for future studies to sample *H. japonica* in a larger scale to verify the presence of polymorphism and to investigate the mechanism that maintains this polymorphism within a population. Potential mechanisms that maintain this polymorphism would be correlational selection^52^, trade-off between survival benefits and either (or both) physiological costs or sexually attractive characters^57^, or other physiological restraints induced by environmental conditions^58^.

### Colour change speed

Most of the colour change process has been completed within an hour and maintained similar levels throughout the day when frogs are against the same coloured background. This corroborates previous studies that demonstrated rapid colour change in frogs^22^,^23^. Rapid colour change to resemble natural backgrounds is pivotal for daily survival, especially when a frogs’ resting place changes within or between days. Since individual *H. japonica* can be found against various background even within a day (A. Borzée, unpublished), rapid colour change ability would be beneficial for individuals that move between substrates frequently.

Interestingly, during the night, frogs became darker but largely retained the colour that they exhibited the previous day in terms of both brightness and chroma (Fig. 6). This could be adaptive if a frog rests on the same background as the previous day because the colour of the frog should already resemble the background in the early morning when many predators are actively searching for prey^59^. In addition, this retaining of colours suggests the possibility of long-term colour change. Since we tested each frog against each background maximally for 12 hours, we do not know about the long-term colour change in *H. japonica* and the upper threshold of dorsal colour change if they stay against one background repeatedly for more than a day. However, previous studies suggest the existence of long-term colour changing in tree frogs. *H. regilla* can undergo slow and gradual colour change during 21 days^26^. In the previous study of *H. japonica*^27^, the background colour matching in terms of brightness and chroma generally and gradually improved over the course of 7 days when the frogs stayed against the same coloured background. Long-term gradual colour change is dominated by morphological colour changing organisms and can be adaptive only when the animals stay against the same background for a longer period. Although mechanisms for long-term colour change in frogs are currently lacking, long-term change could be adaptive if they selectively rest on the same substrate over days.

### Concluding remarks

Camouflage through colour change is a widespread phenomenon in nature. To properly understand this intriguing biological process, it is crucial to consider its evolution under visually heterogeneous environments and the sensory properties of the animals’ natural predators^36^,^60^. Our results provide a new insight on the camouflage function of colour change in anurans and mainly highlight i) background colour matching strategy of tree frogs against visually heterogeneous backgrounds, ii) conditional expression of dorsal patterns, and iii) substantial variation in individual capacity to change colours, and iv) the possibility of polymorphism in terms of hue-changing abilities. Further studies on the fitness benefits of colour change and pattern expression, the relation between colour changing capacity and substrates preference, and the mechanisms that maintain the variation in colour changing capacity and polymorphic morphs within a population would advance our current understandings of colour changing animals.

## Additional Information

**How to cite this article**: Kang, C. *et al*. Colour and pattern change against visually heterogeneous backgrounds in the tree frog *Hyla japonica*. *Sci. Rep*. **6**, 22601; doi: 10.1038/srep22601 (2016).

## Supplementary Material

Supplementary Information

## Figures and Tables

**Table 1:** Median just noticeable differences (JNDs) of frog dorsal colours between different achromatic backgrounds (BK: black; BW: black/white patterned; GY: grey; WH: white).

Comparisons	Bird vision		Snake vision	
	Chromatic JNDs	Achromatic JNDs	Chromatic JNDs	Achromatic JNDs
BK-BW	11.86 (6.18–17.31)	8.54 (6.07–14.21)	4.85 (3.39–8.43)	13.64 (9.63–24.24)
BK-GY	12.51 (7.43–20.90)	11.28 (5.34–19.21)	6.71 (3.00–10.30)	17.98 (8.47–29.65)
BK-WH	14.82 (12.28–25.87)	14.49 (11.70–21.81)	8.03 (5.49–12.38)	22.81 (15.50–35.54)
BW-GY	7.47 (4.82–11.34)	7.19 (4.39–14.19)	4.54 (2.13–7.31)	13.06 (6.06–20.87)
BW-WH	9.08 (5.99–13.41)	9.27 (4.96–17.02)	5.82 (2.70–8.70)	16.34 (7.44–24.97)
GY-WH	8.10 (4.62–10.71)	9.16 (4.14–13.18)	4.19 (1.96–6.78)	11.97 (5.48–19.46)

JNDs from avian predator models were derived using blue tits (*Cyanistes caeruleus*) and snake models were derived from visual pigment absorbance data of garter snakes (*Thamnophis sirtalis*). The ranges in brackets mean first-third quartile ranges (N = 12 for each background).
