# Supplementary material for: Colour and pattern change against visually heterogeneous backgrounds in the tree frog Hyla japonica
Source: Sci Rep. 2016 Mar 2;6:22601. doi: 10.1038/srep22601 (PMC4773871; doi:10.1038/srep22601)
Supplement: Supplementary Information [file srep22601-s1.pdf]

Supplementary Information for the paper:

Colour and pattern change against visually heterogeneous backgrounds in the tree frog, *Hyla japonica*

Changku Kang, Ye Eun Kim, Yikweon Jang

Supplementary Methods: predator vision modelling

Using the linearized and equalized images, we transformed the image's red, green, and blue channel pixel values to a bird-specific colour space (predicted photon catches for each cone cell type) using a polynomial mapping method[1–3] by a custom-built MATLAB program. Camera sensor sensitivities were obtained using quadratic programming methods[4].

Bird vision can be generally categorized into two types: UV-sensitive colour vision (UVS) and Violet-sensitive colour vision (VS)[5]. The main difference between these two types is that Violet sensitive colour vision is less sensitive to ultraviolet wavelengths. Within human visible ranges (400-700 nm), and especially for long and medium wave sensitive cones, the difference between UVS and VS vision is small. Since *H. japonica* reflects low UV and short-wavelength colours (Figure S1), the results from those two models should yield the similar outcomes[3]. Therefore, for bird vision modelling, we used one vision type (UVS visual system) using blue tit (*Cyanistes caeruleus*) as a model species[2,3].

For each pixel, we calculated long-wave-sensitive (LWS), medium-wave-sensitive (MWS), and short-wave-sensitive (SWS) cone photon catches, and double cone photon catches. We assumed UV-sensitive (UWS) cone photon catches zero because both background and frog body reflect very low amount of UV lights. The inputs from single cones are assumed to be used for chromatic discrimination task and the inputs from double cones are assumed to be used for achromatic discrimination task[6]. Then we applied tetra-chromatic receptor-noise-limited visual discrimination model[7,8]. This model produces a colour distance ΔS between two colours as:

$$\Delta S^2 = ((e_1e_2)^2(\Delta f_4 - \Delta f_3)^2 + (e_1e_3)^2(\Delta f_4 - \Delta f_2)^2 + (e_1e_4)^2(\Delta f_3 - \Delta f_2)^2$$

$$+(e_2e_3)^2(\Delta f_4 - \Delta f_1)^2 + (e_2e_4)^2(\Delta f_3 - \Delta f_1)^2 + (e_3e_4)^2(\Delta f_2 - \Delta f_1)^2)/$$

$$((e_1e_2e_3)^2 + (e_1e_2e_4)^2 + (e_1e_3e_4)^2 + (e_2e_3e_4)^2,$$

where Δf_i is the log ratio of the quantum catches of each cone type i, e_i is the standard deviation of the noise in a receptor mechanism given by

$$e_i = v_i/\sqrt{\eta_i}.$$

We assumed photoreceptor (Weber) noise (v_i) for LWS photoreceptors = 0.05[7], the ratio of the number of UWS, SWS, MWS, LWS cone cells (η_i) were set as 1:1.9:2.7:2.7[9]. ΔS predicts signal discriminability as a unit of "just noticeable difference" (JND)[7]. Two colours cannot be discriminated when JND < 1: larger

JND indicates that two colours are more distinct in the receptor space of animals. Achromatic JND was calculated by using double cone photon catches using the following equation[8]

$$\Delta S = |\Delta f_i / e|.$$

For snake vision model, we used visual pigment absorption data of garter snake, *Thamnophis sirtalis*[10]. This is the only data available on the visual systems of diurnal snakes, but the visual systems of diurnal snakes are likely to be conserved among species (personal comm. 2014. E. Loew). ΔS was calculated as:

$$\Delta S^2 = ((e_1)^2(\Delta f_3 - \Delta f_2)^2 + (e_2)^2(\Delta f_3 - \Delta f_1)^2 + (e_3)^2(\Delta f_2 - \Delta f_1)^2 /$$

$$((e_1 e_2)^2 + (e_1 e_3)^2 + (e_2 e_3)^2.$$

The ratio of the number of SWS, MWS, LWS cone cells were set as 1:1.6:7.3[10]. Weber noise for LWS photoreceptors was assumed to be as 0.05 as used in other snake vision studies[11]. Although this choice of Weber noise is arbitrary due to the absence of the demonstrated cases, the changes in Weber noise did not change the results qualitatively. We assumed that LWS cones of snakes were used for achromatic discrimination[10] and calculated achromatic JNDs using the same equation as in the avian model.

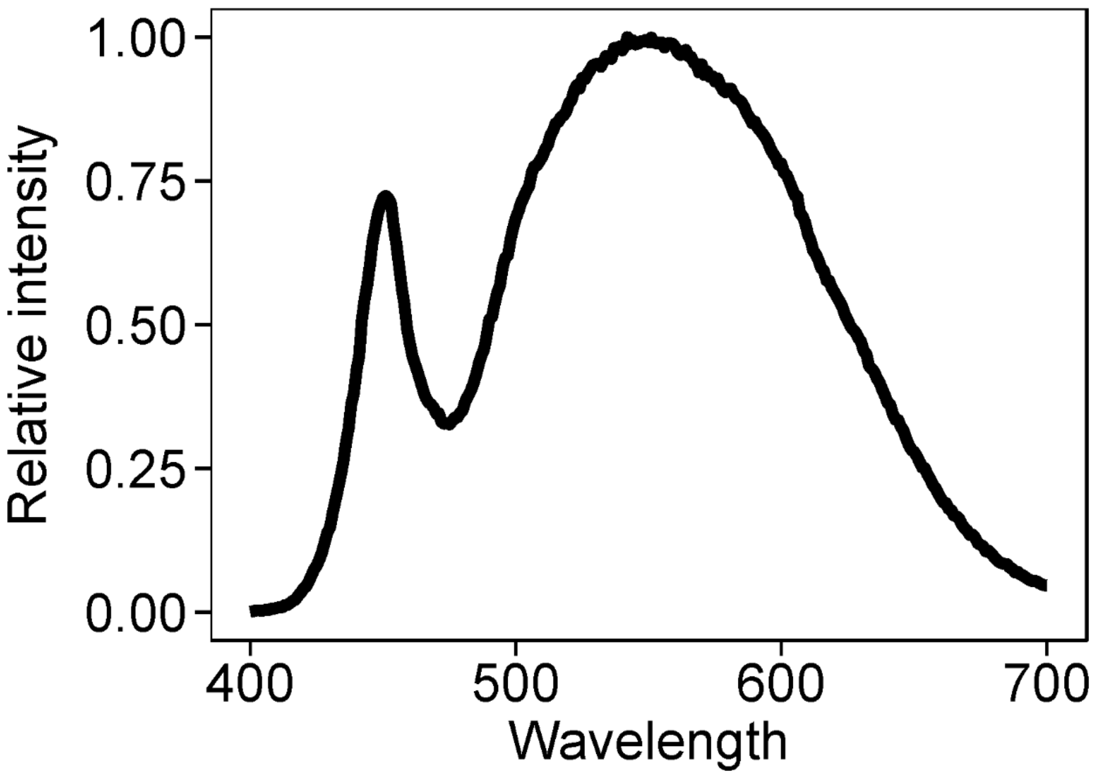

Figure S1. The spectral properties of the LED light source.

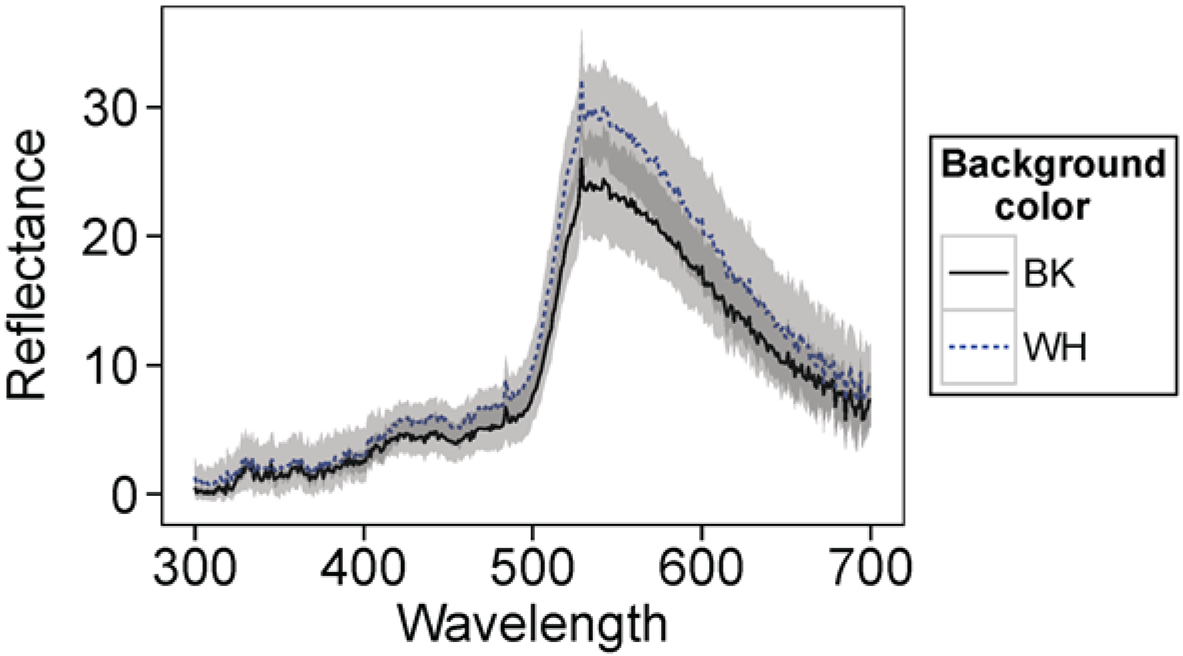

Figure S2. Reflectance of *H. japonica* against BK and WH backgrounds (N=7 for each). Lines denote mean reflectance; shaded areas denote 95% confidence intervals. Frogs reflected negligible amounts of light within UV light regions (between 300-400 nm). Each frog was kept in each background for 2 hours. Spectrometry was used only for checking UV reflection because i) our experimental design required measuring frog colour more than 1,500 times, and ii) handling of frogs can potentially induce colour change.

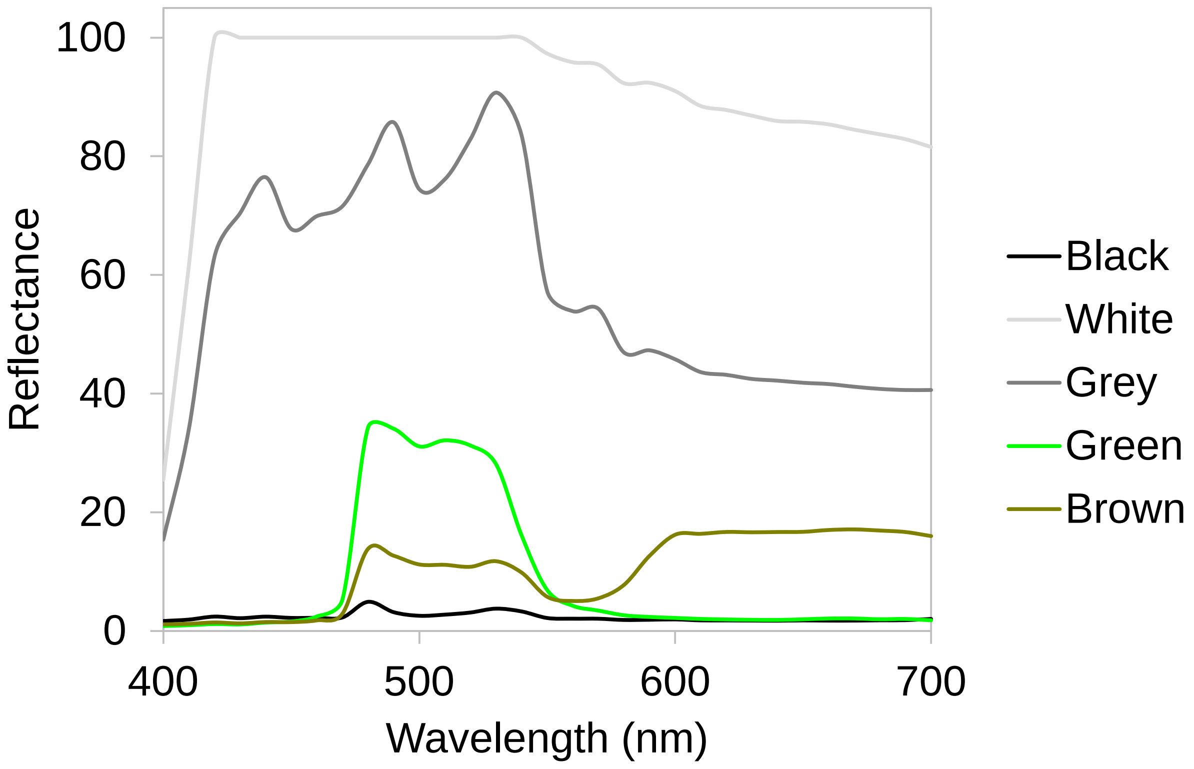

Figure S3. The reflectance of background colours that were used for the experiments.

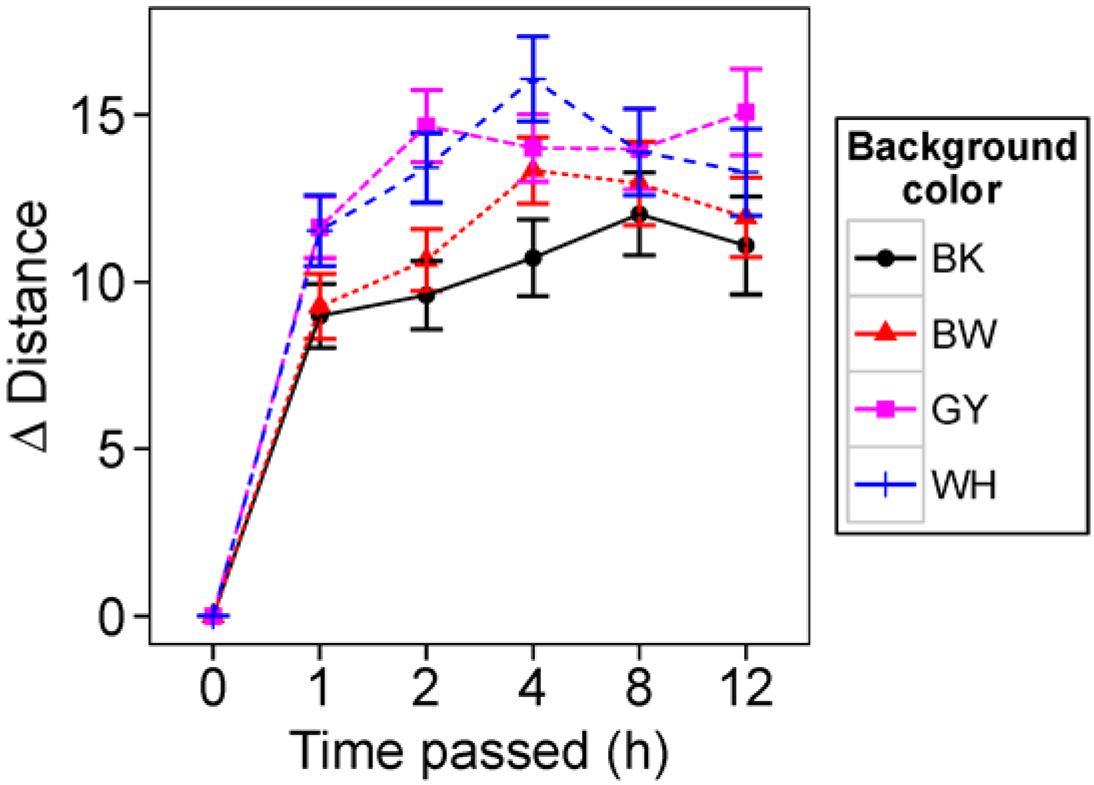

Figure S4. The relationship between time passed since the initiation of each colour changing trial and the Euclidean colour distance (ΔE) from the initial status in BW experiment (N=48). In all four types of backgrounds, frog colours changed abruptly within one hour and maintained similar levels throughout the remaining time. Symbol represent mean values and bars denote standard error of the mean.

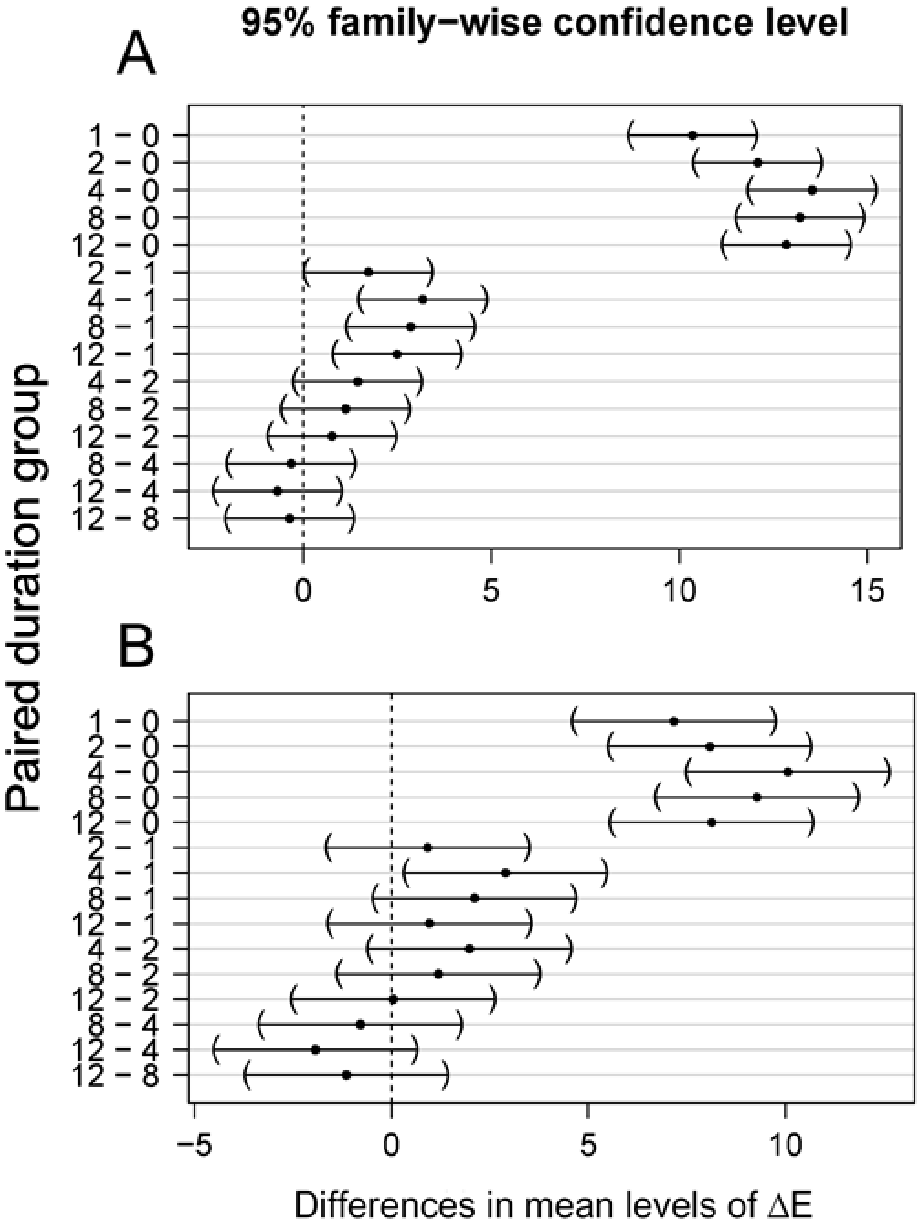

Figure S5. The results of all-pairwise comparisons of ΔE between each time group in (A) BW experiment and (B) GB experiment. Y-axis shows each pair group. Small round circles and range indicate mean difference in ΔE ± 95% family-wise confidence intervals. The colours of frogs after 2 hours were not statistically different from the colours after 1, 4, 8, and 12 hours in both experiments.

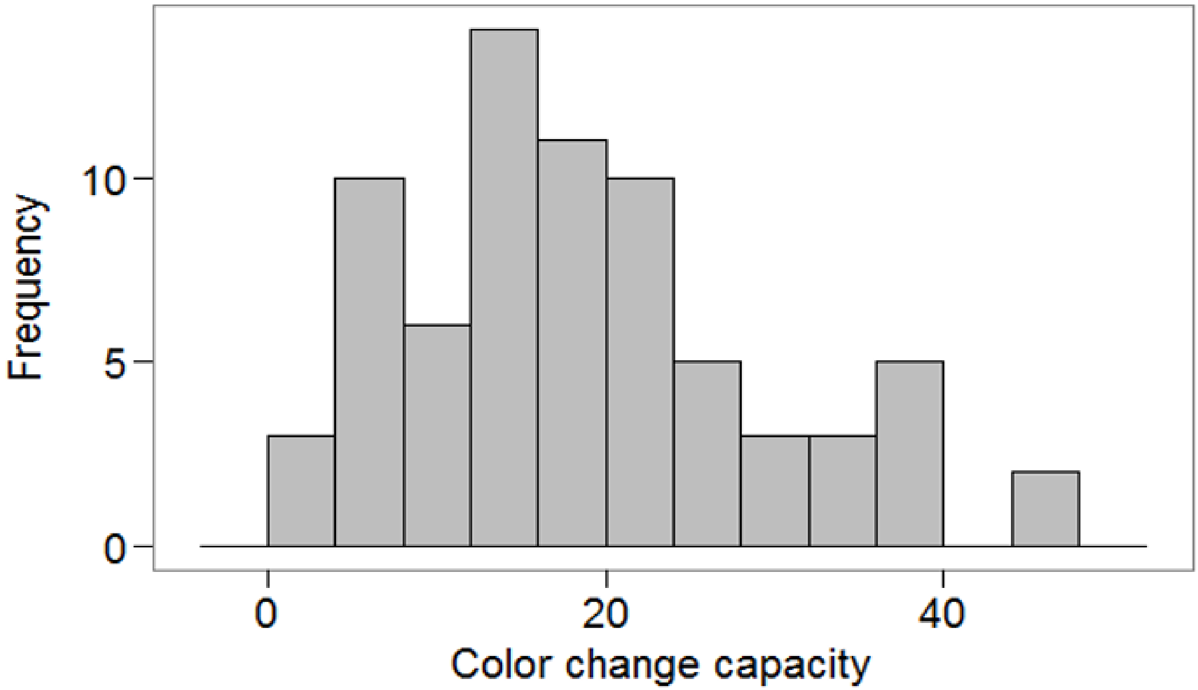

Figure S6. The histogram of colour change capacity of all tested frogs (N=72). The distribution follows unimodal distribution which suggests that colour changing capacity is a continuously varying trait rather than a discrete polymorphic trait.

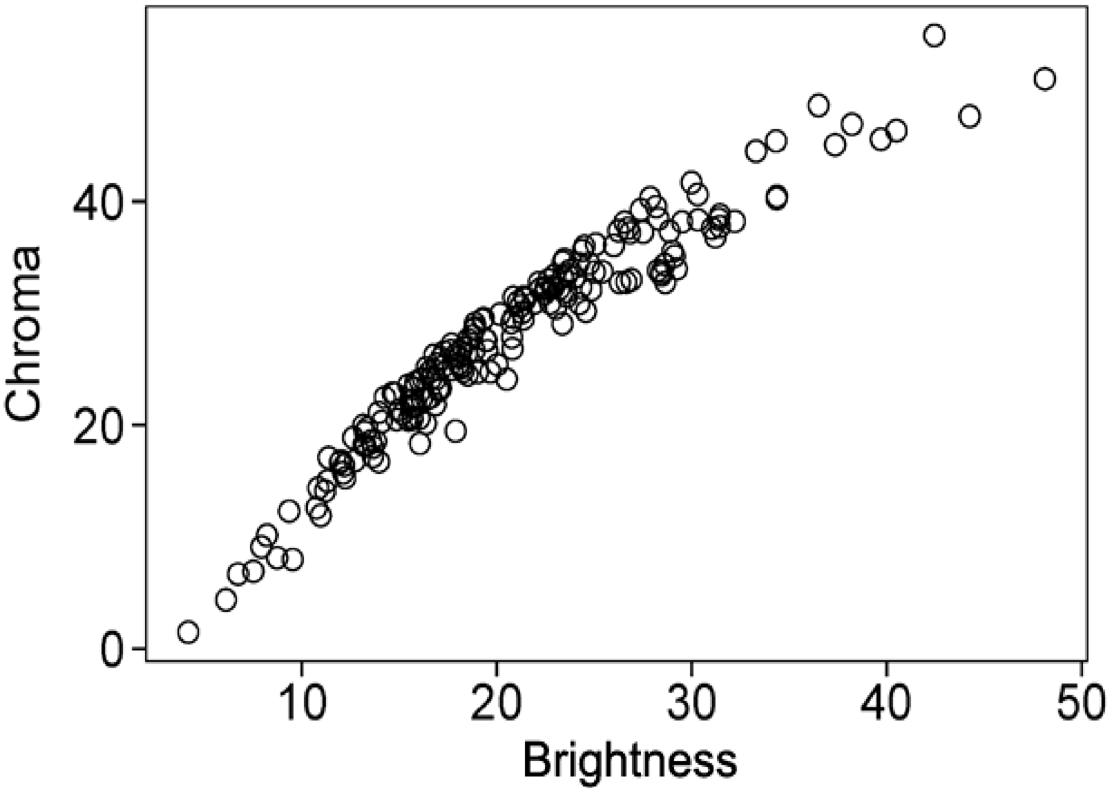

Figure S7. Correlation between brightness and chroma of the dorsal colour of each frog. We found strong positive correlation between brightness and chroma of frog dorsal colours (Pearson's product-moment correlation test; t_{190}=47.47, *r*=0.96, *P*<0.001).

Supplementary results on achromatic JND comparisons

In avian achromatic vision model, estimated JNDs between BK colour and frogs' colour against each background type were lowest for the BK background and increased in the order of BK, BW, GY, and WH, but mean JNDs were not significant different between GY and WH (main text Figure 4; BK-BW: $Z=3.88$, $P<0.001$; BW-GY: $Z=3.76$, $P<0.001$; GY-WH: $Z=2.32$, $P=0.09$). The trend was opposite for GY and WH colour results: JNDs were lowest for the WH background and increased in the order of WH, GY, BW, and BK with non-significant difference between WH-GY (WH-GY: $Z=-2.10$, $P=0.15$; GY-BW: $Z=-3.40$, $P=0.004$, BW-BK: $Z=-4.57$, $P<0.001$).

In snake achromatic vision model, estimated JNDs between BK colour and frogs' colour against each background type were lowest for the BK background and increased in the order of BK, BW, GY, and WH, but mean JNDs were not significant different between GY-WH and between BW-BK (main text Figure 4;BK-BW: $Z=1.43$, $P=0.48$; BW-GY: $Z=3.78$, $P=0.001$; GY-WH: $Z=2.15$, $P=0.14$). The trend was opposite for GY and WH colour results: JNDs were lowest for the WH background and increased in the order of WH, GY, BW, and BK with non-significant difference between WH-GY (WH-GY: $Z=-1.92$, $P=0.22$; GY-BW: $Z=-3.50$, $P=0.002$, BW-BK: $Z=-4.23$, $P<0.001$).

Table S1. The results of post-hoc comparisons between background treatments.

	Brightness		Chroma	
Comparison	Z	P	Z	P
BK - BW	3.61	<0.001	4.57	<0.001
BK - GY	6.95	<0.001	7.91	<0.001
BK - WH	9.08	<0.001	9.98	<0.001
BW - GY	3.34	<0.001	3.34	0.001
BW - WH	5.47	<0.001	5.41	<0.001
GY - WH	2.22	0.03	2.07	0.04

Supplementary References

1. Párraga, C. A., Troscianko, T. & Tolhurst, D. J. Spatiochromatic properties of natural images and human vision. *Curr. Biol.* **12,** 483–487 (2002).

2. Stevens, M., PARraga, C. A., Cuthill, I. C., Partridge, J. C. & Troscianko, T. S. Using digital photography to study animal coloration. *Biol. J. Linn. Soc.* **90,** 211–237 (2007).

3. Kang, C., Stevens, M., Moon, J., Lee, S.-I. & Jablonski, P. G. Camouflage through behavior in moths: the role of background matching and disruptive coloration. *Behav. Ecol.* **26,** 45–54 (2015).

4. Pike, T. W. Using digital cameras to investigate animal colouration: estimating sensor sensitivity functions. *Behav. Ecol. Sociobiol.* **65,** 849–858 (2011).

5. Hart, N. S. & Hunt, D. M. Avian visual pigments: characteristics, spectral tuning, and evolution. *Am. Nat.* **169,** S7–S26 (2007).

6. Osorio, D. & Vorobyev, M. Photoreceptor sectral sensitivities in terrestrial animals: adaptations for luminance and colour vision. *Proc. R. Soc. London B Biol. Sci.* **272,** 1745–1752 (2005).

7. Vorobyev, M. & Osorio, D. Receptor noise as a determinant of colour thresholds. *Proc. R. Soc. London B Biol. Sci.* **265,** 351–358 (1998).

8. Siddiqi, A., Cronin, T. W., Loew, E. R., Vorobyev, M. & Summers, K. Interspecific and intraspecific views of color signals in the strawberry poison frog Dendrobates pumilio. *J. Exp. Biol.* **207 ,** 2471–2485 (2004).

9. Hart, N. S. The Visual Ecology of Avian Photoreceptors. *Prog. Retin. Eye Res.* **20,** 675–703 (2001).

10. Sillman, A. J., Govardovskii, V. I., Röhlich, P., Southard, J. A. & Loew, E. R. The photoreceptors and visual pigments of the garter snake (Thamnophis sirtalis): a microspectrophotometric, scanning electron microscopic and immunocytochemical study. *J. Comp. Physiol. A* **181,** 89–101 (1997).

11. Stuart-Fox, D., Moussalli, A. & Whiting, M. J. Predator-specific camouflage in chameleons. *Biol. Lett.* **4,** 326–329 (2008).
